# Influence of Layer-by-Layer Polyelectrolyte Deposition and EDC/NHS Activated Heparin Immobilization onto Silk Fibroin Fabric

**DOI:** 10.3390/ma7042956

**Published:** 2014-04-11

**Authors:** M. Fazley Elahi, Guoping Guan, Lu Wang, Martin W. King

**Affiliations:** 1 Key Laboratory of Textile Science and Technology, Ministry of Education, College of Textiles, Donghua University, Songjiang District, Shanghai 201620, China; E-Mails: elahitex@yahoo.com (M.F.E.); ggp@dhu.edu.cn (G.G.); 2 College of Textiles, North Carolina State University, Raleigh, NC 27695-8301, USA; E-Mail: martin_king@ncsu.edu

**Keywords:** silk fibroin fabric, layer-by-layer, heparin, EDC/NHS, poly(allylamine hydrochloride), poly(acrylic acid), low molecular weight heparin (LMWH), hemocompatibility

## Abstract

To enhance the hemocompatibility of silk fibroin fabric as biomedical material, polyelectrolytes architectures have been assembled through the layer-by-layer (LbL) technique on silk fibroin fabric (SFF). In particular, 1.5 and 2.5 bilayer of oppositely charged polyelectrolytes were assembled onto SFF using poly(allylamine hydrochloride) (PAH) as polycationic polymer and poly(acrylic acid) (PAA) as polyanionic polymer with PAH topmost. Low molecular weight heparin (LMWH) activated with 1-ethyl-3-(dimethylaminopropyl) carbodiimide hydrochloride (EDC) and N-hydroxysuccinimide (NHS) was then immobilized on its surface. Alcian Blue staining, toluidine blue assay and X-ray photoelectron spectroscopy (XPS) confirmed the presence of heparin on modified SFF surfaces. The surface morphology of the modified silk fibroin fabric surfaces was characterized by scanning electron microscopy (SEM) and atomic force microscopy (AFM), and obtained increased roughness. Negligible hemolytic effect and a higher concentration of free hemoglobin by a kinetic clotting time test ensured the improved biological performance of the modified fibroin fabric. Overall, the deposition of 2.5 bilayer was found effective in terms of biological and surface properties of the modified fibroin fabric compared to 1.5 bilayer self-assembly technique. Therefore, this novel approach to surface modification may demonstrate long term patency in future *in vivo* animal trials of small diameter silk fibroin vascular grafts.

## 1. Introduction

Any biomaterial that is designed and manufactured to be used in direct contact with blood, cannot be expected its application without its surface modification. Undoubtedly, clotting is one of the biggest challenges for a foreign material. Problem of clotting remains a substantial challenge which must be overcome before any material can be used clinically in contact with blood. Especially, when a foreign material is exposed to blood, plasma proteins are absorbed onto the surface, followed by the activation of clotting factors or the adhesion and activation of platelets, and finally the formation of a thrombus [1]. Different chemicals, bioactive molecules, and methods have been developed and investigated in order to obtain a surface that has less complication when in contact with blood. Surface modification by hydrophilic materials or bioactive agents applied either chemically or physically, is the most commonly used techniques to obtain such a surface [2].

There is a great demand for small diameter arterial prostheses with innovative antithrombogenic properties [3]. Even though numerous progresses in biomaterials design and utilization, the perfect artificial small-vessel substitute has yet to be developed. Current commercial synthetic grafts, fabricated from expanded polytetrafluoroethylene (ePTFE) (Goretex^®^, W. L. Gore & Ass. Inc., Newark DE, USA) and polyethylene terephthalate (PET) (formerly Dacron^®^, Boston Scientific, Billerica, MA, USA) are successful for the repair and replacement of the major caliber arteries (inner diameter > 6 mm), but fail for smaller diameters (inner diameter < 6 mm) which are of interest for coronary and femoral artery bypass procedures, due to thrombosis and/or compliance mismatch [4–6] Silk fibroin (SF), a natural protein material is considered an attractive material for biomedical applications [7]. It has excellent biocompatibility, adaptable biodegradability, and good oxygen/water vapor permeability [8]. Recent reports from our own laboratory and others have demonstrated that woven silk fibroin tubular fabrics provide a promising biomaterial for use as a small diameter arterial prosthesis [6,9,10]. Such graft materials fabricated from fibroin fibers provide excellent patency when implanted in the rat abdominal aorta, with 1 year patency rates of 85% which is much higher than for ePTFE (30%) [11]. Unfortunately, pure native silk fibroin is lack of good antithrombogenic property [12] when it’s used as a pure form in contact with blood. Hence, the issue of how to improve the hemocompatibility of small diameter silk fibroin vascular grafts continues to be an important problem to be solved.

In this study low molecular weight heparin (LMWH) was selected as an antithrombogenic bioactive material which is a glycosaminoglycan (GAG) and is extracted primarily from porcine intestinal tissues. It is currently used for the prevention and treatment of venous thrombosis and pulmonary embolism, management of arterial thrombosis in patients presenting with acute myocardial infarction and in the prevention of rethrombosis after thrombolysis [13]. Only 15% to 25% of the chains of LMWH contains the pentasaccharide sequence that is necessary for binding to antithrombin [14]. In contrast, to inactivate thrombin, heparin must bind to both antithrombin and thrombin, thereby forming a ternary complex [15]. This complex can be formed only by pentasaccharide-containing heparin chains composed of at least 18 saccharide units, which is the case for most of the chains of unfractionated heparin but fewer than half of those of low-molecular weight heparins. Thus, LMWH has greater activity against factor Xa than unfractionated heparin, which has equivalent activity against factor Xa and thrombin [16].

In recent years layer-by-layer (LbL) self-assembly of polyanions and polycations into multilayered coatings have been explored for use in a number of technologies, including wetting [17,18], antifogging [19], and biological applications [20–22]. The driving force for this LbL assembly approach is primarily electrostatic interaction, but the process can also involve charge-transfer interactions, van der Waals interactions, hydrogen bonding, and short-range hydrophobic interactions [23,24] between the layer to layer and the layer to substrate phases. One important feature of this method is the adsorption at every step of a polyanion/polycation assembly, which results in recharging of the outermost layer during the fabrication process. The LbL assembly of oppositely charged polyelectrolytes is facilitated by the formation of water-insoluble complexes of poly(allylamine hydrochloride) (PAH) and poly(acrylic acid) (PAA) via the ionic attractions between the carboxylate COO^−^ and ammonium NH_3_^+^ groups [25,26], thereby improving the stability of the polyelectrolyte layers.

In order to improve the hemocompatibility of silk fibroin fabric (SFF), a modification process has been developed. In the first part of modification, 1.5 and 2.5 polyelectrolytes bilayer using PAH and PAA were assembled onto SFF with PAH topmost. PAH solution (pH 4.5) was applied first followed by PAA solution as PAH is positively charged and the silk fibroin surface is negatively charged in acidic condition [27]. After that, EDC/NHS activated heparin was immobilized on PAH layered fibroin fabric surface. According to literature, EDC/NHS can activate carboxyl acid groups of the heparin and promote their covalent binding to amino groups; which has been proved to be non-cytotoxic [28] and biocompatible [29]. Furthermore, the immobilization of heparin to EDC/NHS activated heparin was proved to improve *in vivo* blood compatibility by Wissink *et al.* [30]. It is accepted that heparin carries a net negative charges even at very low pH [31]. Whereas, the PAH carries positive charges and thus heparin and PAH form complexes through electrostatic attraction at appropriate pH values.

Our experimental outcomes suggest that the modification system used in this paper might be an encouraging technique to modify the surface of small diameter arterial prostheses made from silk fibroin. To the best of our knowledge, this is the first study which employs EDC/NHS activated heparin immobilization on polyelectrolytes assembled silk fibroin fabric to improve its hemocompatibility.

## 2. Results and Discussion

For easier understanding and reading of the paper, the sample notation with short description is shown in Table 1. More details can be found in experimental part at the end.

### 2.1. Surface Morphology

**Figure 1.**
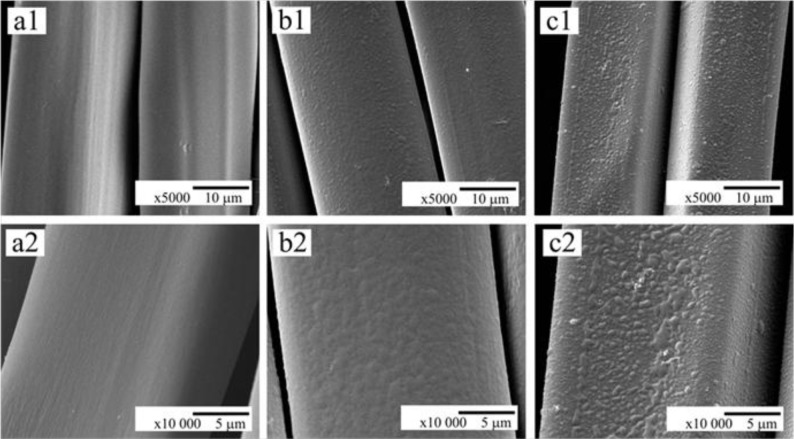
SEM micrographs of untreated and modified fibroin fabrics: (**a1**, **a2**) SFF; (**b1**, **b2**) SFF-1 and (**c1**, **c2**) SFF-2. Magnification: top row 5000×; bottom row 10,000×.

The morphology and topography of the untreated (SFF) and modified fibroin fabrics (SFF-1 and SFF-2) were obtained by SEM and atomic force microscopy (AFM). The surfaces of the modified fibroin materials changed from being smooth [Figure 1(a1,a2)] to being irregular and rough [Figure 1(b1,b2,c1,c2)]. This indicates that the polyelectrolytes bilayer and heparin were not uniformly deposited and distributed on the fabric surfaces. In fact the roughness of the modified fabrics appeared to increase with additional polyelectrolyte layers, because the surface of the SFF-2 sample with 2.5 bilayer of polyelectrolyte was found to be rougher than that of SFF-1. It is possible that more electrolyte bilayers can play a role in increasing the bonding of the heparin layer.

It was noted that on washing in an ultrasonic bath a very limited amount of heparin was released. In fact most of the heparin remained attached to the polyelectrolyte modified surface, which suggests that the immobilization of the activated heparin was successfully achieved onto modified silk fibroin fabric [32]. This may not be simply a case where the increasing number of both positive and negative electrolytes creates more polar radicals and functional groups on the surface which promote stronger binding of the negatively charged heparin molecule. By increasing the thickness of the polyelectrolyte layers, this may provide the heparin molecule with some additional or alternative conformations in which to form and attached to the modified fibroin surface in a lower energy state.

**Figure 2.**
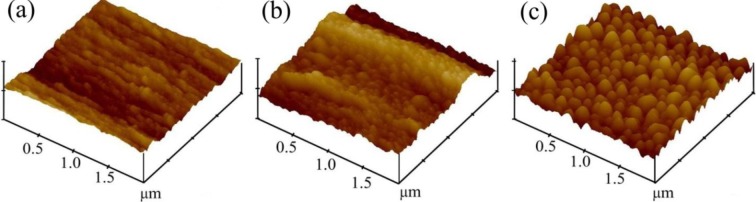
Atomic force microscopy (AFM) images of untreated and surface modified fibroin fabrics: (**a**) SFF; (**b**) SFF-1 and (**c**) SFF-2.

Figure 2 illustrates the typical AFM images taken of the untreated and treated fibroin fabric. The surface topography of fibroin samples appeared to change qualitatively as a result of surface modification. Before modification, the top view of the fibroin surface appeared to be comparatively smooth. After modification, the fiber surfaces became more uneven with many convex protrusions separated by pits or valleys, suggesting that the polyelectrolyte deposition caused irregular condensation clusters on the fiber surface. The quantitative data of the AFM profile expressed as arithmetical mean roughness (Ra) and root mean square roughness (Rq) is shown in Table 2.

### 2.2. ATR-FTIR

**Figure 3.**
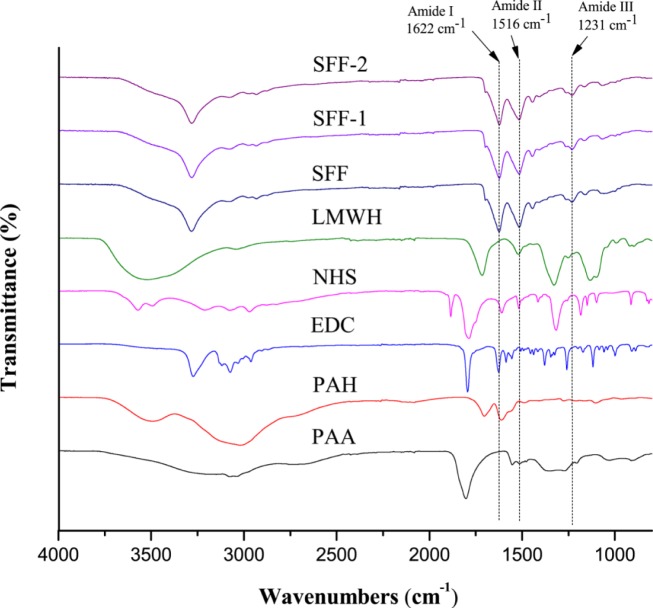
FTIR spectra of poly(acrylic acid) (PAA); poly(allylamine hydrochloride) (PAH); 1-ethyl-3-(dimethylaminopropyl) carbodiimide hydrochloride (EDC); N-hydroxysuccinimide (NHS); low molecular weight heparin (LMWH); SFF, SFF-1 and SFF-2.

FTIR was used to assess any significant changes in the chemistry and fine structure of the fibroin biopolymer material as a result of the polyelectrolyte deposition process. Figure 3 shows the amide I band absorption at 1622 cm^−1^ (~80% CO stretching, ~10% CN stretching, ~10% NH bending vibration), amide II absorption band at 1516 cm^−1^ (~60% NH bending vibration, 40% CN stretching) and amide III band absorption at 1231 cm^−1^ (30% CN stretching, 30% NH bending vibration, 10% CO stretching, 10% O=C–N bending vibration), respectively. The absorption bands are attributed to the β-sheet structure of the silk fibroin [33]. No evident peak shifts were observed between the pure untreated and the surface modified fibroin samples, confirming that there were no conformational changes in the chemical structure of the bulk silk fibroin. These results agree with the findings reported previously on the FTIR analysis of silk scaffolds and vascular grafts containing heparin [34,35]. The sampling depth of the FTIR technique maybe in the order of several microns depending on the wavelength of the incident beam. Positive identification of the nanometer thick polyelectrolyte deposition layers and the EDC/NHS activated heparin layer was therefore not to be expected.

### 2.3. Qualitative and Quantitative Characterization of Heparin

**Figure 4.**
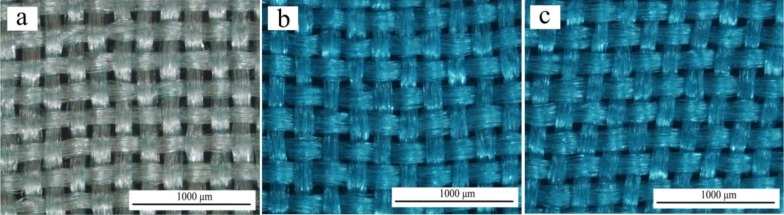
Optical microscopy images of Alcian blue stained untreated and surface modified fibroin fabrics. Magnification: ×40. (**a**) SFF; (**b**) SFF-1 and (**c**) SFF-2.

Alcian Blue is a cationic dye, which can be used to selectively stain anionic glycosaminoglycans (GAGs) such as hyaluronan or heparin. After immobilization of heparin and Alcian Blue staining, an evident difference in color was observed by light microscopy between the untreated (Figure 4a) and treated fibroin fabric surfaces (Figure 4b,c). This result confirms the presence of an active GAG such as heparin and indicates that it is uniformly distributed over the surface of the modified fibroin fabrics. According to the toluidine blue quantitative assay, the heparin content was found to be and 10.65 μg/cm^2^ and 11.03 μg/cm^2^ respectively for the SFF-1 and SFF-2 samples.

### 2.4. Release Test of Heparin

**Figure 5.**
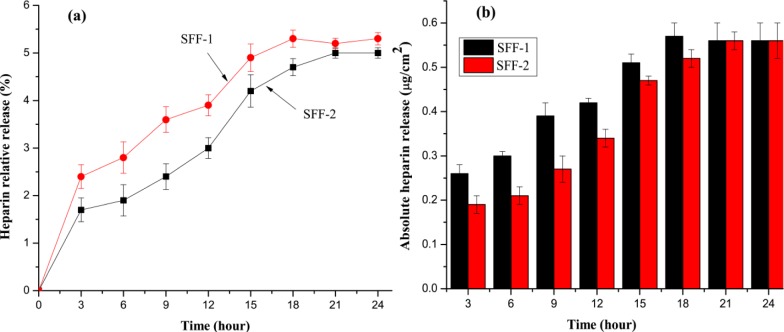
Release percentage of heparin from surface modified silk fibroin fabrics.

Figure 5a shows the release percentage of heparin from the surface modified and heparin immobilized fibroin fabric samples. The amount of heparin released from the samples increased gradually with the duration of immersion time. After 18 h immersion, the maximum release of heparin was found around 5%. After that the curve was stabilized and no release of heparin was observed. The small portion of heparin loss was probably due to non-immobilized physically adsorbed heparin on fibroin fabric surfaces. We also assume that the higher heparin percentage release observed in SFF-1 as the heparin was not attached strongly onto 1.5 bilayer deposited silk fibroin fabric (SFF-1) compared to SFF-2. We consider that the 2.5 bilayer deposited silk fibroin fabric have strong heparin binding capacity than the 1.5 bilayer deposited silk fibroin fabric. The fact that about 95% of heparin immobilized on fibroin fabric could sustain 24 h extraction in PBS solution could be attributed to the bonding between heparin and fibroin fabric via polyelectrolyte assembly. This result is in agreement with the previous reported durability of heparin grafted on blended polyurethane and silk fibroin film [36] and poly(ethylene terephthalate) fabrics [37]. The absolute amount of heparin released (from 3 to 24 h) from SFF-1 and SFF-2 is shown in Figure 5b. The amount of heparin release at each time point was significantly different (*p* > 0.05) between SFF-1 and SFF-2.

### 2.5. XPS Result

**Figure 6.**
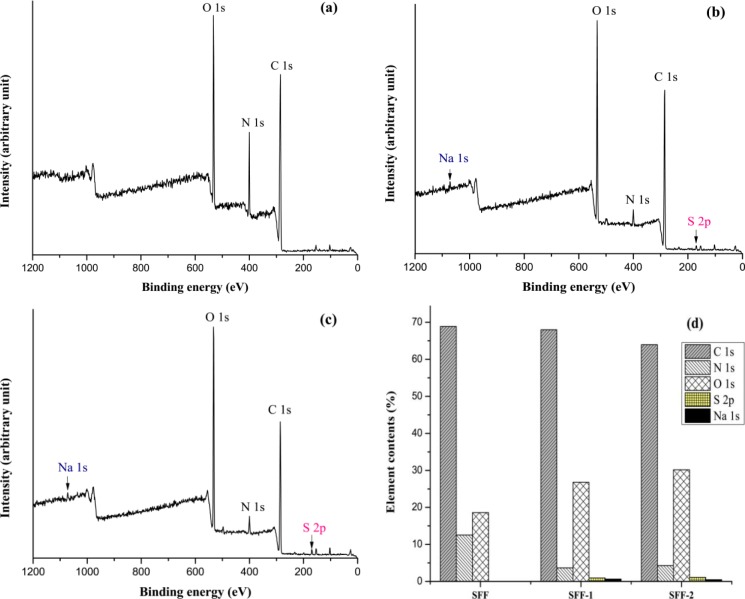
XPS spectra of (**a**) SFF (**b**) SFF-1 and (**c**) SFF-2 and (**d**) relative elemental contents.

**Figure 7.**
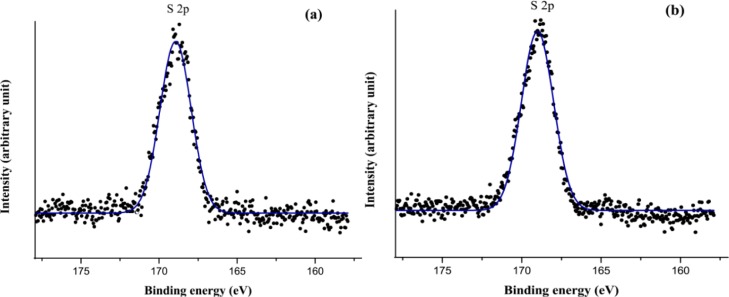
XPS spectra of S 2p peaks: (**a**) SFF-1 and (**b**) SFF-2.

XPS analysis was conducted to evaluate the surface compositions of untreated control SFF, SFF-1 and SFF-2 fabrics with heparin topmost. XPS spectra shows the peaks at the binding energies of 284.73, 400 and 531.59 eV, corresponding to the C 1s, N 1s and O 1s, respectively, with the untreated control SFF, as shown in Figure 6a. With the deposition of polyelectrolytes and immobilization of heparin another two peaks at the binding energies of 168.65 and 1071.25 eV, corresponding to S 2p (sulfur) and Na 1s (sodium) are found (Figure 6b,c), which validated that the heparin was successfully introduced to silk fibroin fabric surfaces. The binding energy peak (168.65 eV) represents sulfur at higher oxidation state assigned to sulfur atoms bonded to two or three oxygen atoms such as sulfone, sulfonate or sulfonic acid [38]. The relative content of O 1s on the modified SFFs compared with SFF is also elevated, which are confirmed by the atomic relative contents shown in Figure 6d. XPS spectra of high resolution S 2p peaks are shown in Figure 7.

The increment of O 1s content and the emergence of S 2p are achieved for the polyelectrolytes deposition and heparin immobilization onto silk fibroin fabric. Further investigation on the chemical structure of the untreated and the modified SFF surface are carried out by the high-resolution XPS analysis of C 1s, O 1s, and S 2p.

**Figure 8.**
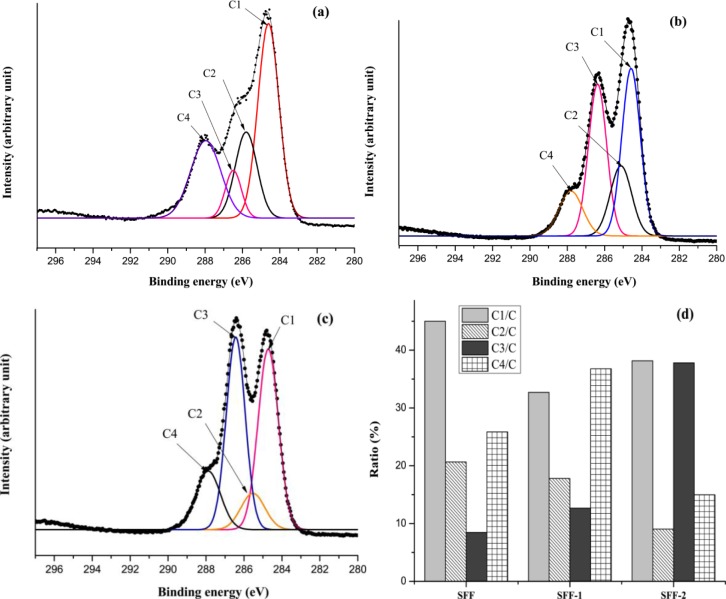
XPS spectra of C 1s peaks (**a**) SFF; (**b**) SFF-1; (**c**) SFF-2 and (**d**) relative elemental contents.

Figure 8a–c gives the XPS spectra of C 1s of untreated and modified SFFs, which show the peaks of C1, C2, C3 and C4, in the range of 284.6, 285.8, 286.5 and 287.9 eV, which were attributed to carbons in –C–H– (and /or –C–C–), –C–O–, C=O (and/or –COOH) and O–C=O groups, respectively, which are consistent with the published data on heparin modified silk fibroin nanofibers [39]. The ratio percentage of carbon elemental content from high-resolution C 1s peaks of silk fibroin fabrics before and after modification has been shown in Figure 8d.

**Figure 9.**
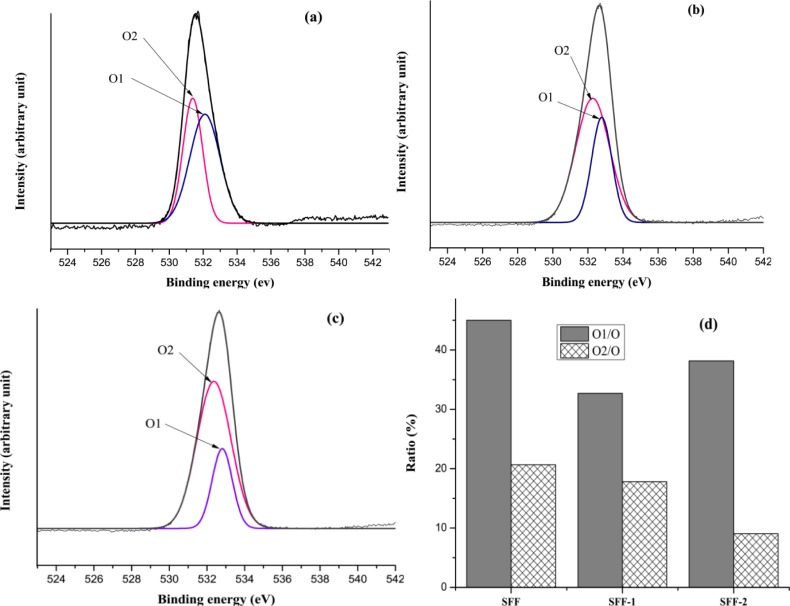
XPS spectra of O 1s peaks (**a**) SFF; (**b**) SFF-1; (**c**) SFF-2 and (**d**) relative elemental contents.

Figure 9a gives the XPS spectra of O 1s of untreated SFF. It shows that the spectrum of SFF consists of O1 and O2 at the range of 531.38 and 532.10 eV, due to the C=O group. On the other hand, the spectrum SFF-1 and SFF-2 consists of O1 and O2 at the range of 532.27–532.36 eV and 532.78–532.8 eV, corresponding to –C–O– group (Figure 9b,c). The detailed oxygen elemental content of the untreated and modified SFFs is presented in Figure 9d.

### 2.6. Hemolytic Effect of Modified Silk Fibroin Fabrics

**Figure 10.**
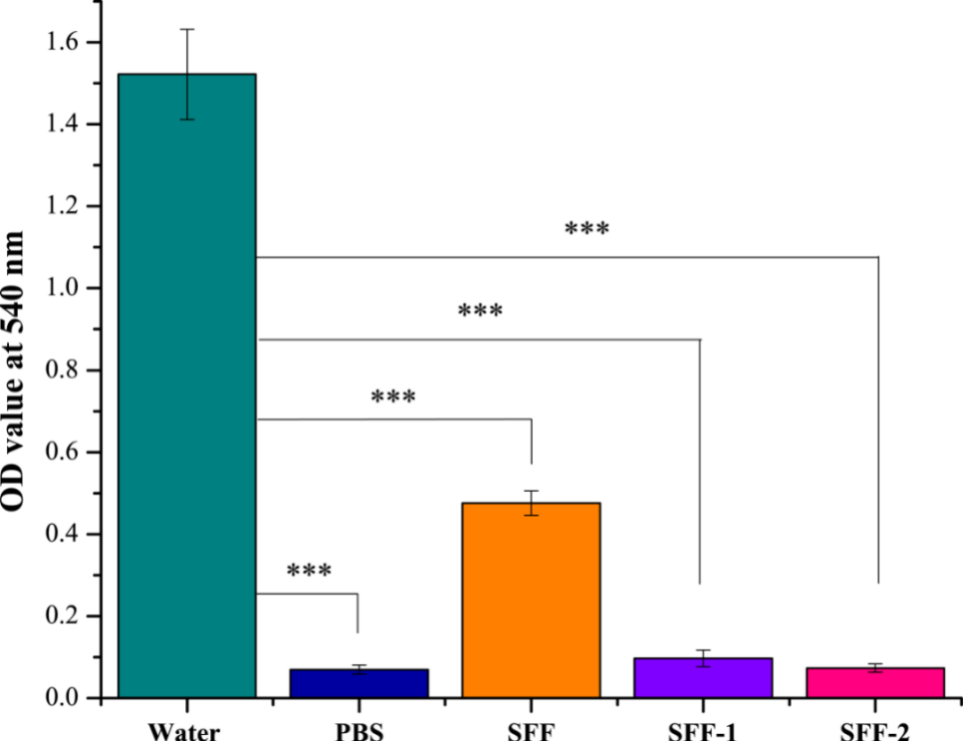
Mean hemolytic assay results of untreated (SFF) and modified fibroin fabric samples (SFF-1 and SFF-2) compared to exposure to the PBS solution negative control and the water positive control. Mean data for each sample (*n* = 3) are presented. Error bar = 1× standard deviation. Statistical differences indicated with (***) for *p* < 0.001.

Hemolysis of the blood is a problem associated with biocompatibility. Red blood cells hemolyze when they come in contact with water. For applications in the artificial small diameter blood vessel, one main concern of the fibrous materials is their potency of hemolysis when contacting blood [40,41]. After exposure of modified fibroin fabric samples to the HRBCs suspension, no obvious hemolytic phenomenon was observed (Figure 10) except the positive control (water). The hemolytic effect of each material was further quantified by recording the absorbance of the supernatant at 540 nm using UV-vis spectroscopy (Perkin Elmer Lambda 25, Waltham, MA, USA). Significant difference (*p* < 0.001) in the OD value can be found between the positive control (water) and the experimental groups (SFF, SFF-1 and SFF-2). Significant difference (*p* < 0.001) in OD value is also observed between SFF to negative control (PBS, 0.01 M, pH 7.4). However, no significant difference (*p* > 0.05) is observed between SFF-1 to SFF-2 and PBS.

The hemolytic index is a direct measure of free hemoglobin present in plasma after exposure to a given material or stressor. An isotonic solution (PBS) served as the negative control and distilled water as the corresponding positive control, inducing osmotic stress that ruptures red blood cells. ASTM standard (ASTM F756-00, 2000) classifies the material as non-hemolytic (0%–2% of hemolysis), slightly hemolytic (2%–5% of hemolysis) and hemolytic (>5% of hemolysis). HP (%) value of SFF is found higher than modified samples (SFF-1 and SFF-2) and significant difference (*p* < 0.05) can be found between SFF to SFF-1, SFF to SFF-2 and SFF-1 to SFF-2. Our results (Table 3) showed that heparin modified silk fibroin fabrics could be classified, as according to the standard, as non-hemolytic while; untreated fibroin fabric as hemolytic.

### 2.7. Rate of Coagulation Property of Modified Silk Fibroin Fabrics

**Figure 11.**
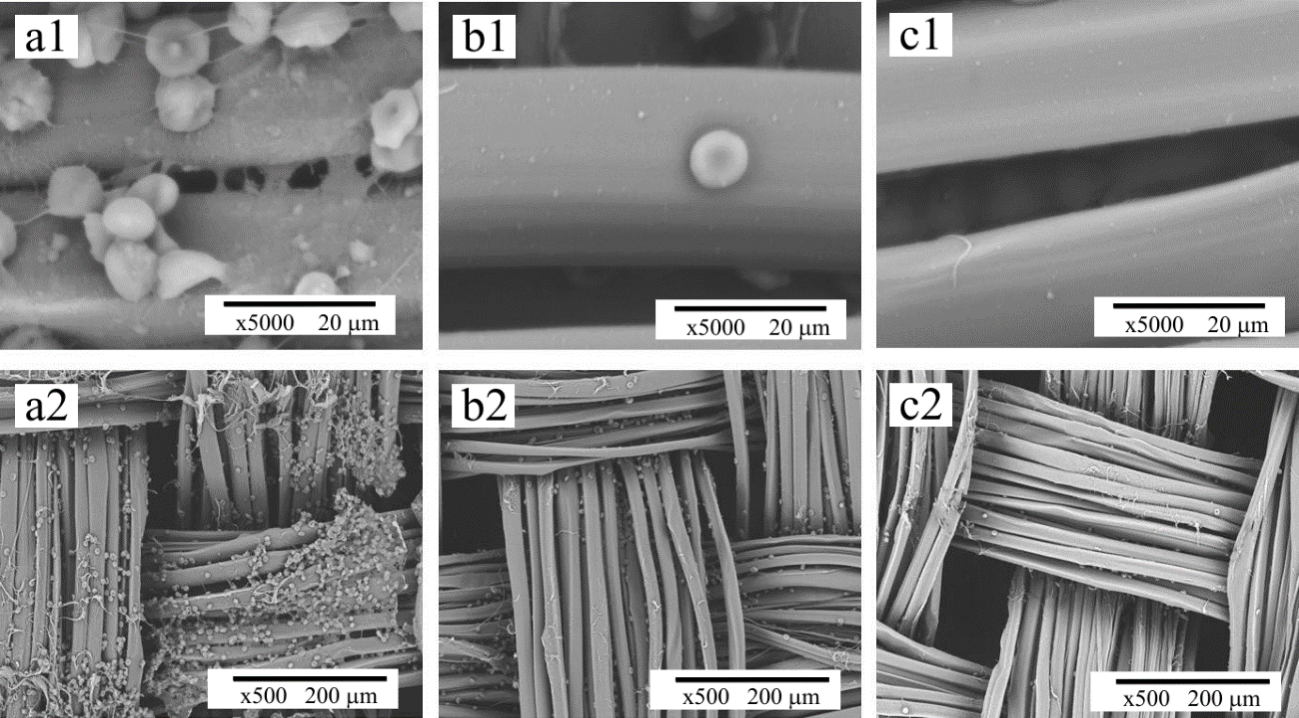
SEM photomicrographs of untreated and surface modified fibroin fabrics after exposure to whole blood: (**a1**, **a2**) Untreated SFF sample showing cell attachment; (**b1**, **b2**) SFF-1 and (**c1**, **c2**) SFF-2 surface treated samples show limited and no cell attachment respectively. Magnification: top row 5000×; bottom row 500×.

The coagulation of blood is initiated by thrombin that transforms fibrinogen into fibrin monomer, which under normal conditions forms long chain fibrin fibers, resulting in a stabilized clot or thrombus network containing red blood cells, white blood cells and activated platelets [42]. The red blood cells that are trapped within a fibrin clot will not release their hemoglobin as rapidly as those mobile cells that are not part of the thrombus. As a result the rate of thrombus formation can be monitored indirectly by measuring the concentration of free hemoglobin in the diluted blood sample following incubation and clotting for specific periods of time. Therefore, to evaluate the applicability of a biomaterial to be used in contact with blood, it is important to investigate the rate of this coagulation cascade on the biomaterial. SEM images of untreated and modified fibroin fabric samples after exposure to whole blood are presented in Figure 11. They clearly show that the surface of the unmodified fibroin fabric (SFF) was covered with accumulated blood cells, while that of the surface modified samples (SFF-1 and SFF-2) showed almost no evidence of cellular attachment. This limited *in vitro* experiment suggests that the heparin immobilized fibroin fabrics are associated with a slower clotting process.

**Figure 12.**
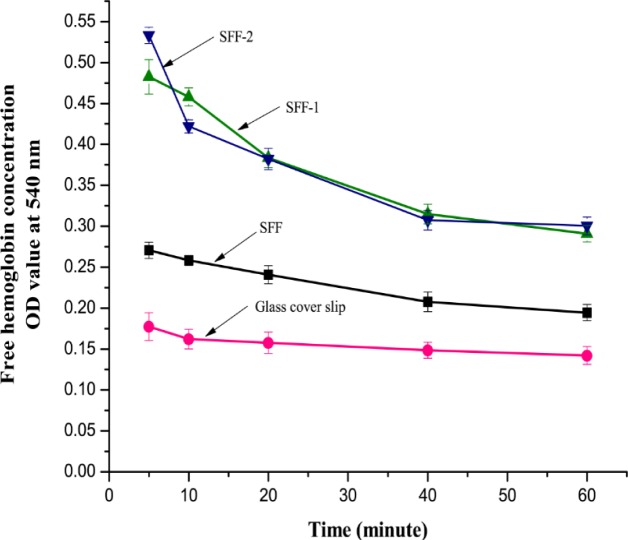
Rate of coagulation assay of untreated and surface modified silk fibroin fabrics at different time intervals. Data are shown here as mean (n = 3). Error bar = 1 × standard deviation.

Figure 12 shows the mean optical density (OD) of free hemoglobin in the supernatant diluted blood following coagulation for different periods of time and exposure to distilled water. A higher OD value represents a higher hemoglobin concentration and suggests that the rate of clotting associated with the material is slower. The OD values of the modified fibroin fabrics are relatively higher at each time point than those of the untreated fibroin fabric and the glass cover slips. This may be due to the fact that the heparin is well incorporated within the fibroin fiber when compared with that of the untreated fibroin fabric. In contrast, under similar experimental conditions, glass cover slips displayed significantly faster clotting behavior after incubation for 60 min. The absorbance of hemoglobin in the case of glass cover slip was 0.14, which is the lowest value because glass is one of the most thrombogenic materials [43]. These results revealed that all the modified fibroin fabrics possessed slower rates of coagulation than the unmodified fibroin samples. The enhanced anticoagulant property by heparin immobilization could be explained from the negative charge density on the silk fibroin surfaces. Heparin immobilization increased negative charge density of the fibroin fabric surfaces, which may have played a role in inhibiting the activities of some clotting factors of blood plasma.

## 3. Experimental Section

### 3.1. Materials

**Figure 13.**
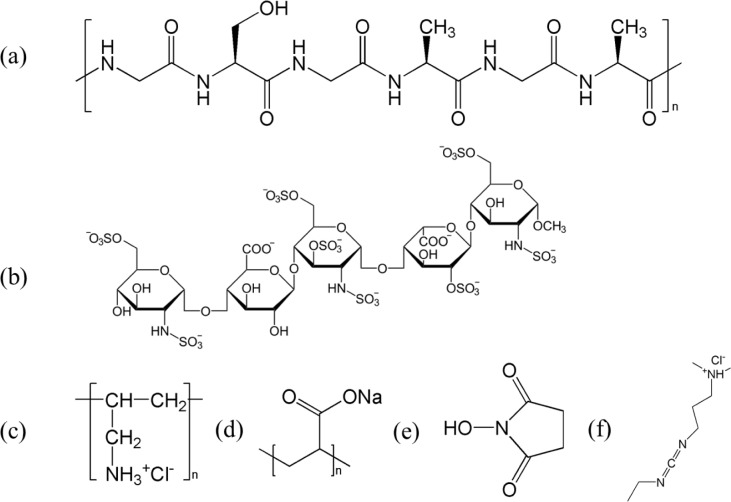
Chemical structures of the materials used: (**a**) silk fibroin; (**b**) LMWH with two typical GAG repeats with multiple sulfate groups; (**c**) PAH; (**d**) PAA; (**e**) EDC and (**f**) NHS.

Plain structured pure 100% silk (*Bombyx mori*) fabric derived from silk filaments supplied by Soho International Silk Company Ltd, Jiangsu Province, China, was used as the base material for preparation of untreated and modified silk fibroin fabric. Sodium carbonate (Na_2_CO_3_) was purchased as a powder from Biochemical Technology Company Ltd, Shanghai, China. Picric acid was supplied by Xilong Chemical Company Ltd, Shantou, China; and carmine by China National Medicines Company Ltd (Shanghai, China). The calcium chloride (CaCl_2_) used for the hemocompatibility test was purchased from Merck. Fetal bovine serum (FBS) was bought from Gibco (Paisley, UK). Phosphate buffered saline (PBS, 0.01 M pH 7.4) was bought from Solarbio (Beijing, China). According to the supplier of PBS, 0.01 M refers to the concentration of PO_4_^3−^ in PBS solution. PBS was bought as a solid mixture of different salts. The concentration of different salts in the mixtures, according to the supplier was: Na_2_HPO_4_·12H_2_O = 0.008 mol/L; NaH_2_PO_4_·2H_2_O = 0.002 mol/L and NaCl = 0.137 mol/L. Low molecular weight heparin (LMWH) (sodium salt, CAS 9041-08-1, ≥ 130 U/mg) and toluidine blue (CAS 92-31-9) were purchased from Sinopharm Chemical Reagent Co., Ltd. Shanghai. The 1-ethyl-3-(dimethylaminopropyl) carbodiimide hydrochloride (EDC) and N-hydroxysuccinimide (NHS) were procured from Shanghai Medpep. Co., Ltd. The poly (allylamine hydrochloride) (PAH) (average Mw = 58000), and poly(acrylic acid) (PAA) (average Mw = 1800), the Alcian blue 8GX and bovine serum albumin (BSA) used for heparin staining were purchased from Sigma-Aldrich. The polyelectrolytes were used as received without further purification and were prepared as 1 g/L solutions. Figure 13 shows some structures of the materials used to perform the experiments.

### 3.2. Preparation of Untreated Fibroin Fabrics

Pure 100% as received silk fabric was degummed by treating 3 times in 0.05% (w/w) Na_2_CO_3_ solution at 98 °C to remove the sericin gum. Each degumming process lasted 30 min, followed by washing in distilled water and being allowed to dry at room temperature. After degumming, the fabric was dyed with combined picric acid-carmine dye liquor. This compound dye stains fibroin yellow and sericin red. Observing the color generated on the silk fabrics before and after degumming, we were able to confirm the effectiveness and uniformity of the degumming process.

### 3.3. Formation of Polyelectrolytes Bilayer on Silk Fibroin Fabric Surfaces

**Figure 14.**
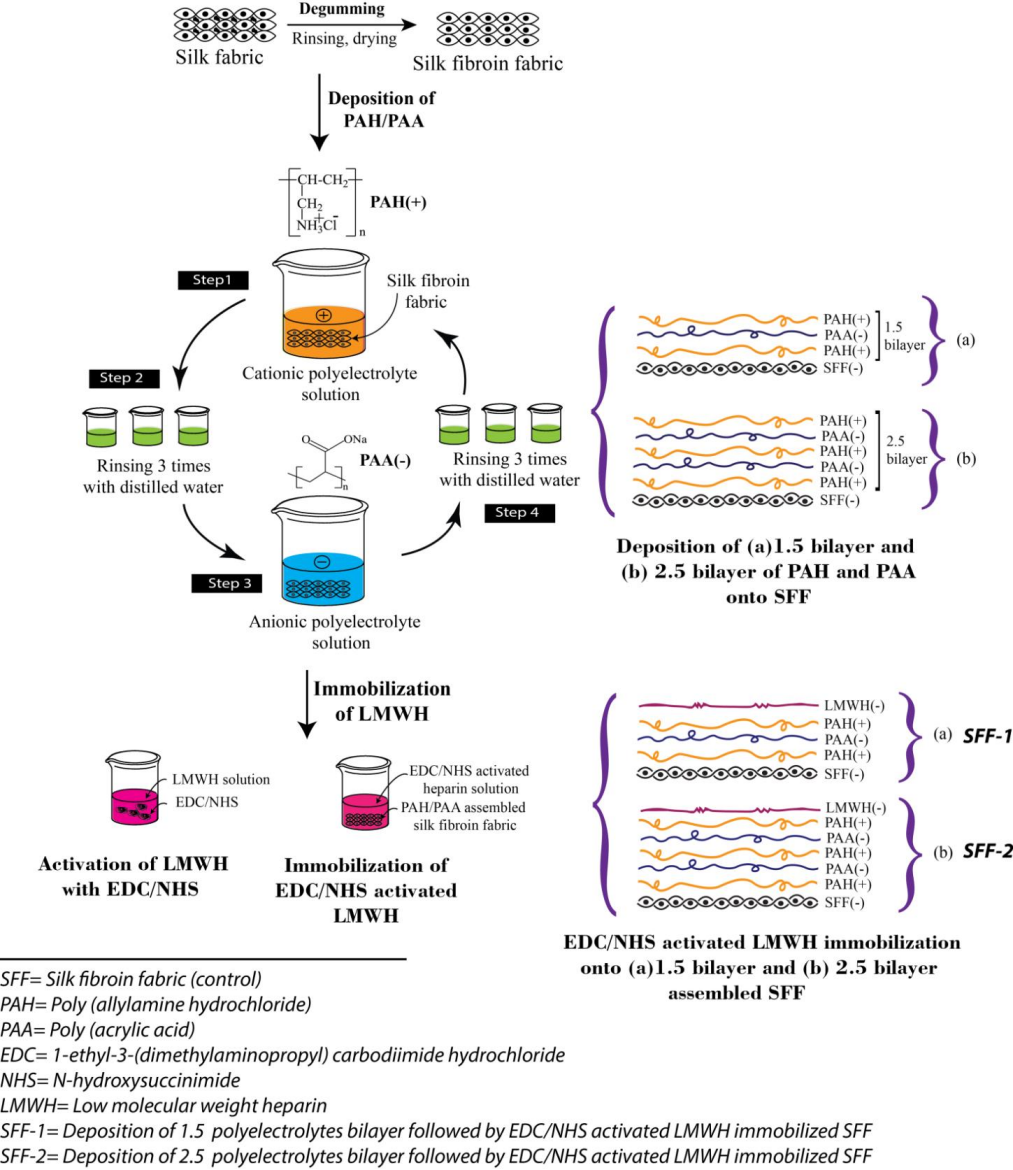
Schematic illustration of the deposition of poly(allylamine hydrochloride) (PAH) and poly(acrylic acid) (PAA) to create 1.5 and 2.5 polyelectrolytes bilayer on silk fibroin fabric (SFF) surfaces followed by EDC/NHS activated heparin immobilization.

The schematic illustration of polyelectrolytes deposition by LbL technique onto SFF has been shown in Figure 14. The 1.5 and 2.5 polyelectrolytes bilayer on fibroin fabric surface were generated by sequentially dipping the substrates into aqueous solutions of poly(allylamine hydrochloride) (PAH) and poly(acrylic acid) (PAA). Both PAH and PAA solutions were prepared at a concentration of 1 mg/mL by dissolving the electrolytes in distilled water. Then the fibroin fabric was first immersed in the PAH solution and incubated at 37 °C and 100 rpm for 30 min followed by rinsing 3 times, each time for 1 min in a distilled water bath. The fabric was then immersed in the PAA solution for 30 min followed by the same rinsing steps in triplicate. The electrostatic adsorption and rinsing steps were repeated until the desired number of deposition bilayer (1.5 and 2.5) was obtained and the outermost layer was PAH. These modified silk fibroin fabrics covered by the polyelectrolyte bilayers were dried at room temperature for 24 h prior to the subsequent heparin immobilization. Due to unavailability of the profilometry/ellipsometry, we couldn’t measure the thickness of bilayer. Moreover, measurement of the layer thickness on a textile surface is difficult because of its inherent irregular surface [44]. However, a thickness of 20–25 nm/bilayer is expected as observed from other paper [45].

### 3.4. Immobilization of EDC/NHS Activated Heparin onto Polyelectrolytes Assembled Fibroin Fabric Surfaces

EDC/NHS solution was prepared using PBS (0.01 M, pH 7.4) and stirred well in an ultrasonic cleaner for 15 min. The concentration of the LMWH solution was fixed as 1 mg/mL by dissolving the powder in PBS (0.01 M, pH 7.4) solution. The solution of EDC/NHS was then mixed with the heparin solution and maintained the pH of 5.7 using PBS (0.01 M, pH 7.4) in order to activate the carboxylic acid groups of heparin. The modified fibroin fabrics were allowed to react with the EDC/NHS activated heparin solution at 4 °C for 24 h. After the immobilization reaction, the modified fibroin fabrics were washed with PBS and then rinsed with distilled water in an ultrasonic cleaner for 10 min.

### 3.5. Characterization Techniques

The surface morphology of the untreated and modified samples was observed by SEM (Quanta-250, FEI, Delmont, PA, USA) and AFM (Nanoscope IV, Veeco Instruments Inc., Plainview, NY, USA). The attenuated total reflectance-Fourier transform infrared spectroscopy (ATR-FTIR) of untreated and modified SFF was conducted on a Nicolet 6700 (Thermo Fisher Scientific Inc., Waltham, MA, USA) FTIR spectrometer over the range of 750–4000 cm^−1^. X-ray Photoelectron Spectroscopy (XPS) test was performed using ESCALAB MKII spectrometer (VG Scientific, Montreal, QC, Canada). Measurements of elemental contents are based on the average of several measurements (Table 4).

### 3.6. Qualitative Characterization of Heparin

Alcian Blue staining was used to identify the presence of any heparin retained on the substrate surfaces [5]. Briefly, the untreated (SFF) and surface modified silk fibroin fabrics (SFF-1 and SFF-2) were first treated separately with 10 mg/mL bovine serum albumin (BSA) in PBS (0.01 M, pH 7.4) at 37 °C for 1 h and then rinsed with PBS three times. Samples were then stained with Alcian Blue 8GX (2%, w/V) diluted with 3% acetic acid solution at 37 °C for 30 min and washed five times with distilled water. Finally, the degree of color intensity of the surface modified and untreated fibroin fabrics was observed by optical microscopy (Nikon, Otawara, Japan).

### 3.7. Quantitative Characterization of Heparin

The amount of heparin (LMWH) immobilized on the polyelectrolyte deposited fibroin fabrics was determined quantitatively by the toluidine blue method reported by Park *et al.* [46]. According to this paper, a standard curve of heparin using a range of heparin solution (5–35 mg/mL) was created to measure the amount of heparin deposited on silk fibroin fabric. 2 mL heparin solution was first mixed with 3 mL of toluidine blue solution (25 mg toluidine blue dissolved in 500 mL 0.01 M HCl containing 0.2% NaCl) and was shaken well to ensure the complete reaction. After 30 min, 3 mL n-hexane was added to the solution, mixed, and allowed to phase-separate. The heparin/toluidine blue complex which formed was extracted with the n-hexane and its concentration was determined using a UV-visible spectrophotometer (Perkin Elmer Lambda 25) at 631 nm. The standard curve for relating the absorbance measurement to the concentration of heparin was then established. For measuring the amount of heparin immobilized on the surface of the fibroin fabrics, 2 mL of distilled water and 3 mL of toluidine blue solution were added into an empty 10 mL conical flask. Samples of the heparin immobilized fibroin fabric measuring 1 cm × 1 cm were then immersed in the solution and the reaction was allowed to continue for 30 min. After that, 3 mL of n-hexane were added to the flask, which was shaken to accelerate the extraction of the heparin/toluidine blue complex by the n-hexane. By measuring the absorbance of the aqueous solution at 631 nm, the amount of heparin on the surface of the fibroin fabric samples was quantified. This analysis was undertaken in triplicate and the average results are presented.

### 3.8. Release Test of Heparin from Modified Silk Fibroin Fabrics

SFF-1 and SFF-2 samples (1 cm × 1 cm) in triplicate were immersed in a beaker containing 50 mL PBS (0.01 M, pH 7.4) at room temperature for 24 h. Two pieces of samples were taken out at predetermined times (*t* = 3, 6, 9, 12, 18, 19, and 24 h) and the amount of heparin remained on those samples were determined using the toluidine blue method. The release of heparin can be calculated as the Equation (1) [37].

$$\text{Release of heparin}\;(\%)=\frac{\text{D}1-\text{D}2}{\text{D}1}\times 100\%$$ (1)

where, D1 and D2 are the surface densities of LMWH on modified silk fabrics before and after extraction with PBS solution, respectively.

### 3.9. Hemolytic Assay

Human whole blood anti-coagulated with 3.8% sodium citrate solution, with a human whole blood to aqueous solution ratio of 9:1 V/V, was kindly provided by Shanghai First People’s Hospital (Shanghai, China). In order to completely remove the serum, the blood was centrifuged and washed with PBS (0.01 M, pH 7.4) five times using a Biofuge Primo Model R centrifuge following standard procedures reported in the literatures [47,48]. The separated red blood cells were first of all suspended by diluting 35 times in PBS solution. That means that 1 mL of human red blood cell (HRBC) was added with 34 mL PBS Then 0.2 mL of the HRBC suspension was transferred to a 5 mL Eppendorf tube (Eppendorf AG, Hamburg, Germany) which was filled with either 0.8 mL of deionized water as the positive control or PBS as the negative control. Both the untreated and surface modified fibroin fabric samples were incubated in the suspension containing 0.2 mL diluted HRBC and 0.8 mL PBS at 37 °C for 2 h, followed by centrifugation (10,000 rpm; 3 min) in an Eppendorf 5415 Model R centrifuge (Eppendorf AG). Then the optical density of the supernatant at 540 nm was determined by a UV-visible spectrometer (Perkin Elmer Lambda 25). The hemolysis percentage (HP) was calculated using Equation (2) [49,50].

$$\text{HP }(\%)=\frac{{\text{D}}_{\text{t}}-{\text{D}}_{\text{nc}}}{{\text{D}}_{\text{pc}}-{\text{D}}_{\text{nc}}}\times 100\%$$ (2)

where, D_t_ is the absorbance of the test sample; D_pc_ and D_nc_ are the absorbance’s of the positive and negative controls, respectively.

### 3.10. Rate of Coagulation Assay

The rate of coagulation of the untreated and modified fibroin fabrics was determined by a kinetic clotting time method that has been described previously [51,52]. To perform this assay, the untreated and modified fibroin fabric samples were cut into small squares measuring 1.5 × 1.5 cm^2^ in triplicate and were put into individual wells in a 12-well tissue culture plate. Glass cover slips were added to some of the wells as the positive control. Then anticoagulated whole human blood (20 μL) was dropped onto the surface of the fabric samples and the glass cover slip controls. In order to start the blood coagulation cascade, 10 μL of CaCl_2_ solution (0.2 mol·L^−1^) was added to the blood in each well and incubated at 37 °C for one of the predetermined periods of time, either 5, 10, 20, 40 or 60 min. Then 5 mL distilled water was carefully placed in each well and incubated at 37 °C for 5 min. The concentration of free hemoglobin in the aqueous blood solution was measured by determining the optical density at 540 nm using UV-visible spectrophotometer (Perkin Elmer Lambda 25). In preparation for SEM, the samples were fixed in 2% buffered glutaraldehyde solution in a refrigerator at 4 °C for 30 min, dehydrated by a series of solutions with increasing ethanol concentration (*i.e.*, 55%, 70%, 80%, 90%, 95% and 100%) and then dried in a desiccator. Before viewing in the SEM the samples were coated with gold-palladium during a 60 s sputter-coating procedure. The thickness of the gold-palladium coating on the samples for SEM could be very few nanometer (2–5 nm) and hence it doesn’t influence the surface morphology of the material.

### 3.11. Statistical Analysis

The data are reported as means and standard deviations, and the error bars in the figures correspond to 1 x standard deviation. All the statistical analyses were performed using a one way analysis of Variance (ANOVA) statistics. The *p* value of <0.05 was selected as the confidence interval where differences were first found to be significant. The data in the tables are indicated with (*) for *p* < 0.05, (**) for *p* < 0.01, and (***) for *p* < 0.001.

## 4. Conclusions

In the present study, EDC/NHS activated low molecular weight heparin was immobilized onto polyelectrolytes assembled silk fibroin fabric in order to investigate its surface characteristics and to enhance its hemocompatibility. The surface modification was achieved by self-assembly deposition of 1.5 and 2.5 polyelectrolytes bilayer on the silk fibroin fabric surfaces. SEM and AFM showed that the deposition of 2.5 bilayer followed by heparin immobilization generated a rougher surface topography compared to the 1.5 bilayer technique. Alcian Blue staining and toluidine blue assay confirmed the successful immobilization of heparin on the silk fibroin fabric surfaces. In addition to this, X-ray photoelectron spectroscopy ensured the presence of sulfur and sodium atom which was originally sourced from low molecular weight heparin molecule. The incorporation of EDC/NHS activated heparin resulted an acceptable hemolysis percentage (<5%) and a higher concentration of free hemoglobin following the use of a kinetic clotting time assay. These data indicate that the modified and heparin immobilized fibroin fabrics displayed good hemocompatibility. In addition, the present study has pointed out that the deposition of a 2.5 bilayer of PAH and PAA is more effective than a 1.5 bilayer, for enhancing the hemocompatibility and releasing less heparin. To the best of our knowledge, this is the first time that the use of a polyelectrolyte layer-by-layer surface modification technique has been applied to silk fibroin fabrics, and combined with EDC/NHS activated heparin immobilization. Future work will include other hemocompatibility tests such as platelet adhesion test, activated partial thromboplastin time (aPTT) test, and cytocompatibility assays.

## Figures and Tables

**Table 1.** Sample notation and description.

Sample ID	Sample description
SFF	Untreated silk fibroin Fabric (control)
SFF-1	Deposition 1.5 polyelectrolytes bilayer followed by EDC/NHS activated heparin immobilized silk fibroin fabric
SFF-2	Deposition of 2.5 polyelectrolytes bilayer followed by EDC/NHS activated heparin immobilized silk fibroin fabric

**Table 2.** Surface roughness values of untreated and modified silk fibroin fabrics determined from AFM analysis.

Samples	*R*_a_ (nm)	*R*_q_ (nm)
SFF	9.44	11.92
SFF-1	25.38	31.89
SFF-2	56.35	66.20

**Table 3.** Hemolysis percentage of untreated and modified silk fibroin fabrics.

Samples	Hemolysis percentage
SFF	7.31 ± 1.27
SFF-1	1.19 ± 0.50
SFF-2	0.26 ± 0.10

**Table 4.** Measurements of elemental contents.

Samples	Frequency of measurements				
	Na 1s	S 2p	O 1s	N 1s	C 1s
SFF	10	10	3	3	3
SFF-1	6	5	3	3	3
SFF-2	6	5	3	4	3
